# First report of *Aedes albopictus* infected by Dengue and Zika virus in a rural outbreak in Brazil

**DOI:** 10.1371/journal.pone.0229847

**Published:** 2020-03-12

**Authors:** Helder Ricas Rezende, Camila Malta Romano, Ingra Morales Claro, Giovana Santos Caleiro, Ester Cerdeira Sabino, Alvina Clara Felix, Jefferson Bissoli, Sarah Hill, Nuno Rodrigues Faria, Theresa Cristina Cardoso da Silva, Ana Paula Brioschi Santos, Crispim Cerutti Junior, Creuza Rachel Vicente

**Affiliations:** 1 Núcleo de Entomologia e Malacologia, Secretaria de Estado da Saúde do Espírito Santo, Serra, Espírito Santo State, Brazil; 2 Hospital das Clínicas HCFMUSP (LIM52), Faculdade de Medicina, Universidade de São Paulo, São Paulo, São Paulo State, Brazil; 3 Departamento de Moléstias Infecciosas e Parasitárias, Faculdade de Medicina, Universidade de São Paulo, São Paulo, São Paulo State, Brazil; 4 Instituto de Medicina Tropical de São Paulo, Universidade de São Paulo, São Paulo, São Paulo State, Brazil; 5 Vigilância em Saúde, Secretaria Municipal de Saúde de Linhares, Linhares, Espírito Santo State, Brazil; 6 Department of Zoology, University of Oxford, Oxford, United Kingdom; 7 Vigilância em Saúde, Secretaria de Estado da Saúde do Espírito Santo, Vitória, Espírito Santo State, Brazil; 8 Departamento de Medicina Social, Programa de Pós-Graduação em Doenças Infecciosas, Universidade Federal do Espírito Santo, Vitória, Espírito Santo State, Brazil; Fundacao Oswaldo Cruz Instituto Rene Rachou, BRAZIL

## Abstract

In Brazil, Dengue (DENV) and Zika (ZIKV) viruses are reported as being transmitted exclusively by *Aedes aegypti* in urban settings. This study established the vectors and viruses involved in an arbovirus outbreak that occurred in 2019 in a rural area of Espírito Santo state, Brazil. Mosquitoes collected were morphologically identified, sorted in samples, and submitted to molecular analysis for arboviruses detection. Phylogenetic reconstruction was performed for the viral sequence obtained. All 393 mosquitoes were identified as *Aedes albopictus*. DENV-1 genotype V was present in one sample and another sample was positive for ZIKV. The DENV-1 clustered with viruses that have circulated in previous years in large urban centers of different regions in Brazil. This is the first report of *A*. *albopictus* infected by DENV and ZIKV during an outbreak in a rural area in Brazil, indicating its involvement in arboviral transmission. The DENV-1 strain found in the *A*. *albopictus* was not new in Brazil, being involved previously in epidemics related to *A*. *aegypti*, suggesting the potential to *A*. *albopictus* in transmitting viruses already circulating in the Brazilian population. This finding also indicates the possibility of these viruses to disperse across urban and rural settings, imposing additional challenges for the control of the diseases.

## Introduction

Dengue virus (DENV) and Zika virus (ZIKV) are etiological agents of reemerging and emerging infectious diseases that constitute important global public health concerns [1]. Both are RNA viruses belonging to the *Flaviviridae* family, genus *Flavivirus*, that are transmitted to humans by the bite of infected mosquitoes of the *Aedes* genus (*Stegomya* subgenus) [2]. Consequently, DENV and ZIKV present an epidemiological overlap, with occurrence influenced by similar environmental and socioeconomic characteristics, having the same geographical distribution and seasonality [3].

In 2019, more than 100 countries were endemic for DENV and 87 had evidence of autochthonous transmission of ZIKV [4]. Brazil is currently the nation with the highest report of DENV [5] and ZIKV infections in the world [4]. There, *Aedes aegypti* is the only species proven to be involved in the transmission of these viruses [5–7]. Consequently, epidemics affect mainly Brazilian urban areas, due to the adaptation of the vector to this environment [7].

*Aedes albopictus* was never associated with DENV and ZIKV transmission in Brazil, despite its recognition as a competent vector for at least 22 pathogens [8, 9], with proven capacity to transmit both viruses [7, 9–12]. In this country, *A*. *albopictus* is present in all regions, including at least 59% of municipalities [13] and more than 24 states [14]. There, this mosquito is typically present in areas with dense vegetation but has colonized consistently anthropic areas [10, 15]. Brazilian studies have described *A*. *albopictus* presence in urban areas with reminiscent forests [5, 6, 13, 16–22], in slums [15], in suburban areas [13, 16], and in peridomicile and intradomicile location [16, 23], evidencing its dispersion across sylvatic and urban settings [10, 15] and its domestication [15]. The ability to use natural and artificial containers of water for breeding [9, 12, 15–18, 21, 24] supports this ecological plasticity [25].

In Espírito Santo state, Brazil, this vector has been reported since the 1980s, with a broad dispersion in its territory, mainly in areas with underbrush [26]. Ports of this state were presumed as the first entry places of *A*. *albopictus* in Brazil [13, 27]. There, 73,998-suspected cases and 37 deaths by DENV infection were reported in 2019 until the 39° epidemiological week, and 1,055 registers of ZIKV were made in the same period [28].

The introduction of an arbovirus in an area with the presence of vectors must be treated as a relevant event [2]. In March 2019, an outbreak of dengue-like illness with 20-suspected cases of DENV infection was reported in a rural area of Linhares municipality, in Espírito Santo state, Brazil. This study investigated this unexpected autochthonous rural occurrence, establishing the vectors and viruses possibly involved in the transmission.

## Materials and methods

### Study location

**Fig 1:**
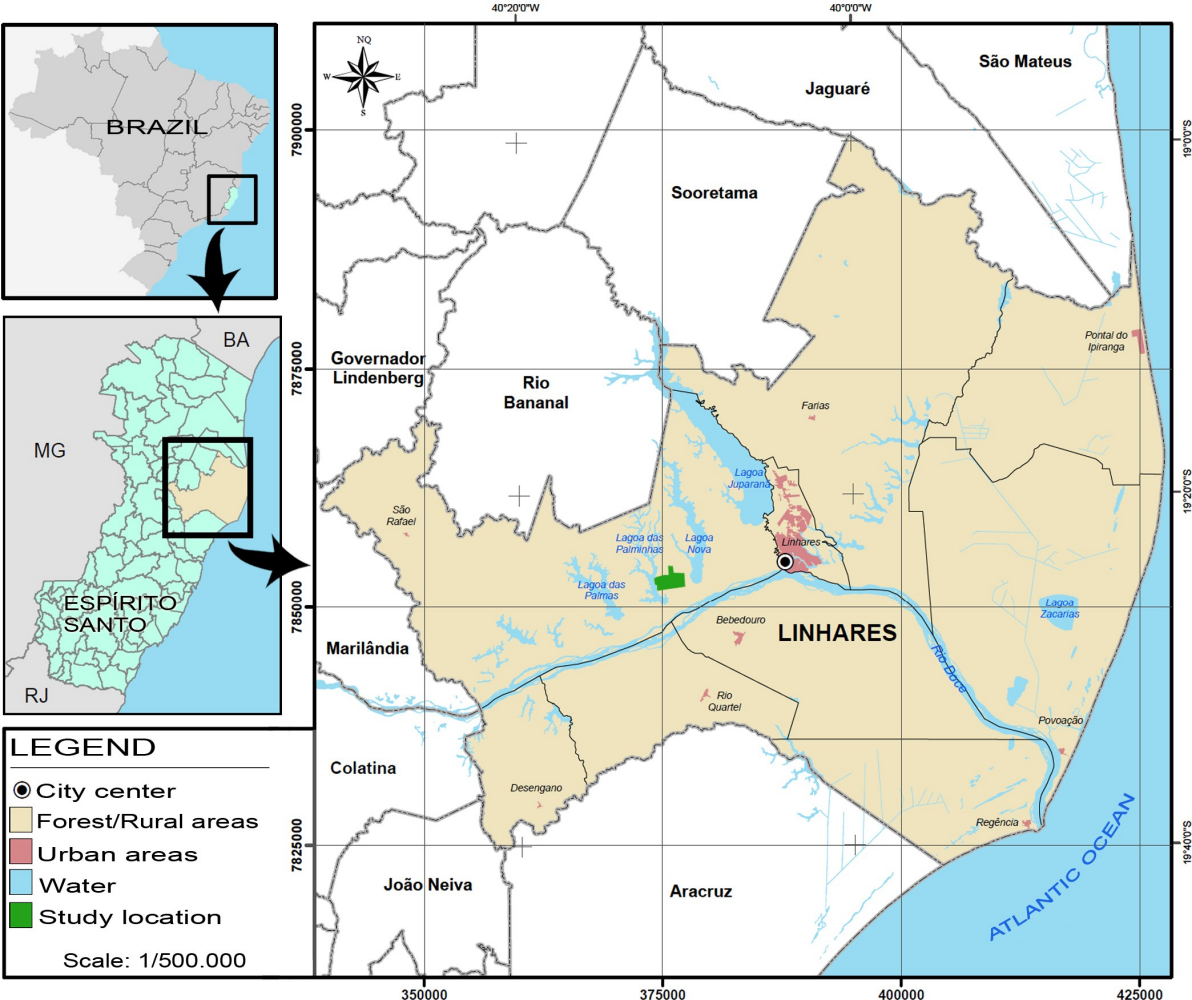
Study location. The abbreviations correspond to the name of the following Brazilian states: Bahia (BA), Minas Gerais (MG), Rio de Janeiro (RJ). Open source map modified from https://geobases.es.gov.br/mapas-munic%C3%ADpios-es.

The mosquitoes were collected in a farm located at 13.6 km distance from the center of Linhares municipality, in the North of Espírito Santo state, Brazil (19°24'38.46'' S, 40°10'13.22'' W). The farm has an area of 463.6 hectares, with 61 hectares of Atlantic Rainforest and approximately 214.6 hectares of cocoa plantation. Two lakes are adjacent to the farm: Lagoa Nova and Lagoa das Palminhas. There were 20 brick houses constructed in the farm headquarters, where 38 people live (Fig 1).

### Epidemiological scenario

During the outbreak, there were 20 reports of exanthematous febrile illness. Four cases were tested for DENV infection by the public health authority: one was positive in the NS1 and viral isolation tests, confirming DENV-1 circulation, and three were positives in the Elisa IgM test. Ten cases were not tested but had confirmation by clinical epidemiological criteria. None infection was reported as caused by ZIKV. Nevertheless, six cases tested were negative and presented inconclusive diagnoses. None of the patients has reported recent travel. The attack rate of this rural outbreak, considering all 20 symptomatic cases and 38 local inhabitants, was 52%.

### Insects sampling

The mosquitoes were collected on 26, 27 and 28th March 2019 at different times of day by using entomological net (sweeping) and insect aspirator (Castro model) [29] in intradomicile, peridomicile and in a cocoa and rubber tree plantations located within a radius of 24 meters from the residences.

### Specimen identification

The mosquitoes were identified based on morphological characters using the identification keys from Consoli and Oliveira (1994) [30] under a stereomicroscope (Olympus SZ61).

### Molecular analysis

After the entomological identification, the mosquitoes were stored in cryogenic tubes with guanidine isocyanate, aiming for the preservation of the genetic material in order to verify the natural infection of the mosquito by the viruses. Mosquitoes were divided into subsamples with pools of approximately 10 to 15 individuals per tube according to the date and time of collection. Numbers and letters, according to the sample code and subsample number of origin, identified the subsamples. For instance, the second pool from sample code 1 was named 1b (Table 1).

**Table 1:** Sampling description.

Sample code	Day	Time	Number of mosquitoes	Number of subsamples (name of the subsample: #mosquitoes per sample)
1	26 March 2019	1:30 pm– 4:00 pm	35	3 (a, b, c: #10) 1 (d: #5)
2	27 March 2019	8:30 am– 10:30 am	110	4 (a, b, c, d: #15) 5 (e, f, g, h, i: #10)
3	27 March 2019	2:30 pm– 4:00 pm	185	9 (a, b, c, d, e, f, g, h, i: #15) 5 (j, k, l, m, n: #10)
4	28 March 2019	9:30 am– 11:00 am	63	1 (a: #13) 5 (b, c, d, e, f: #10)

The mosquitoes were macerated in FastPrep-24 5G Instrument (MP Biomedicals, Ohio, USA) in 1 mL of phosphate-buffered saline solution with 0.75% bovine albumin. Viral RNA was extracted using the QIAamp Viral RNA Mini Kit following the manufacturer’s instructions (QIAGEN, Hilden, Germany).

Molecular tests for arboviruses detection were made through Real-Time PCR. Dengue detection was made according to Huhtano et al. (2010) [31] and serotype determination was accessed using primers and probes serotype-specific described by Callahan et al. (2001) [32]. Zika RNA detection was done using the protocol developed by Lanciotti et al. (2008) [33] employing their second set of primers and probe (ZIKV-1086/1162c). Finally, chikungunya Real-Time PCR was also done using the protocol described by Cecilia et al. (2015) [34].

The evaluation of the presence of human DNA in possibly engorged mosquitoes was performed by using primers and probes directed to RNaseP, according to the protocol described by the World Health Organization [35].

### Multiplex tiling PCR

The extracted RNAs that were positive for DENV-1 and ZIKV were submitted to whole-genome amplification using a tiling, multiplex PCR approach that has been previously developed [36]. Briefly, the sample was converted to cDNA using random hexamers (Invitrogen; Carlsbad CA, USA) and ProtoScript II Reverse Transcriptase (New England BioLabs; Ipswich, MA, USA) according to the manufacturer's instructions. The cDNA was then amplified with a multiplex PCR assay designed from Primal Scheme using as input the “ZikaAsian” scheme for ZIKV (https://github.com/zibraproject) and the one described by Quick et al. (2017) for DENV-1 [36]. An 80% consensus generated from a reference alignment of DENV-1 sequences was used as input to Primal Scheme. PCR was performed using the Q5 High-Fidelity DNA polymerase (NEB). PCR products were cleaned-up using a 1:1 ratio of AMPure XP beads (Beckman Coulter, Brea, CA) and quantified using fluorimetry with the Qubit dsDNA High Sensitivity Assay on the Qubit 3.0 instrument (Life Technologies).

### Nanopore library preparation and sequencing

MinION libraries were generated using the EXP-NBD104 (1–12) Native Barcoding and SQK-LSK109 Kits (Oxford Nanopore Technologies, Oxford, UK). Libraries were loaded onto an FLO-MIN106 flowcell on the MinION device (Oxford Nanopore Technologies) and sequenced using MinKNOW 1.15.1 with the standard 48-hour run script.

### Bioinformatics workflow

Raw files were basecalled using Guppy software version 2.2.7 GPU basecaller (Oxford Nanopore Technologies), then demultiplexed and trimmed by Porechop version 0.2.4 (https://github.com/rrwick/Porechop). Demultiplexed fastQ files were then inputted in CLC Genomics Workbench 6 (CLC Bio, Qiagen). First, the reads were trimmed to remove short (below 50) and low-quality reads (default parameter). Using a DENV-1 genome sampled in Brazil as reference (GenBank ID KP188543), reads were assembled using the following parameters: mismatch cost = 1, indels cost = 2, length fraction that must match reference = 0.8 and similarity fraction = 0.8. A total of 83.8% of reads were correctly mapped to the DENV-1 reference genome, and a consensus sequence of 8413 nucleotides was generated.

The sequence obtained in the study was deposited in GenBank under the accession number MN567709.

### Phylogenetic analysis

The envelope gene of the DENV-1 consensus obtained here by deep sequence was then aligned to globally sampled DENV-1 genomes from all five genotypes using the method implemented in the CLC Genomics Workbench. A dataset containing 93 sequences with 1,485 nucleotides (complete envelope region) was used to infer a maximum likelihood phylogenetic tree in PhyML implemented in SeaView v.4 [37], with 1,000 nonparametric bootstrap replicates. The GTR+I was used as the best nucleotide substitution model as chosen by the jModelTest [38].

### Permissions

The mosquitoes sampling was performed as part of the standard procedures of the Environmental Surveillance Service by the local health authority with landowner permission.

The Information System for Notifiable Diseases (SINAN) was used to access the epidemiological information. Ethical approval was not required since all data accessed retrospectively were aggregated and anonymized.

## Results

A total of 393 mosquitoes were collected in four samples, all identified as *A*. *(Stegomyia) albopictus* (Skuse, 1894) and females. ZIKV was amplified in the subsample 2f (subsample f of sample 2). The presence of the human Cytb gene in the subsample 2f suggests that one or more mosquitos in such a pool were engorged. Subsample 3i (subsample i of sample 3) was positive for DENV-1, and no human DNA was detected in it (Table 2).

**Table 2:** Positive results on RT-PCR.

Subsample code	Day	Time	Virus	Ct
2f	27 March 2019	8:30 am– 10:30 am	ZIKV	35
3i	27 March 2019	2:30 pm– 4:00 pm	DENV	23.7

Fourteen reads of the ZIKV gene were amplified and sequenced, resulting in a consensus sequence with 401 nucleotides, and 98.8% similarity with the ZIKV genome. Due to the low sequence coverage, phylogenetic analysis was not performed for ZIKV.

**Fig 2:**
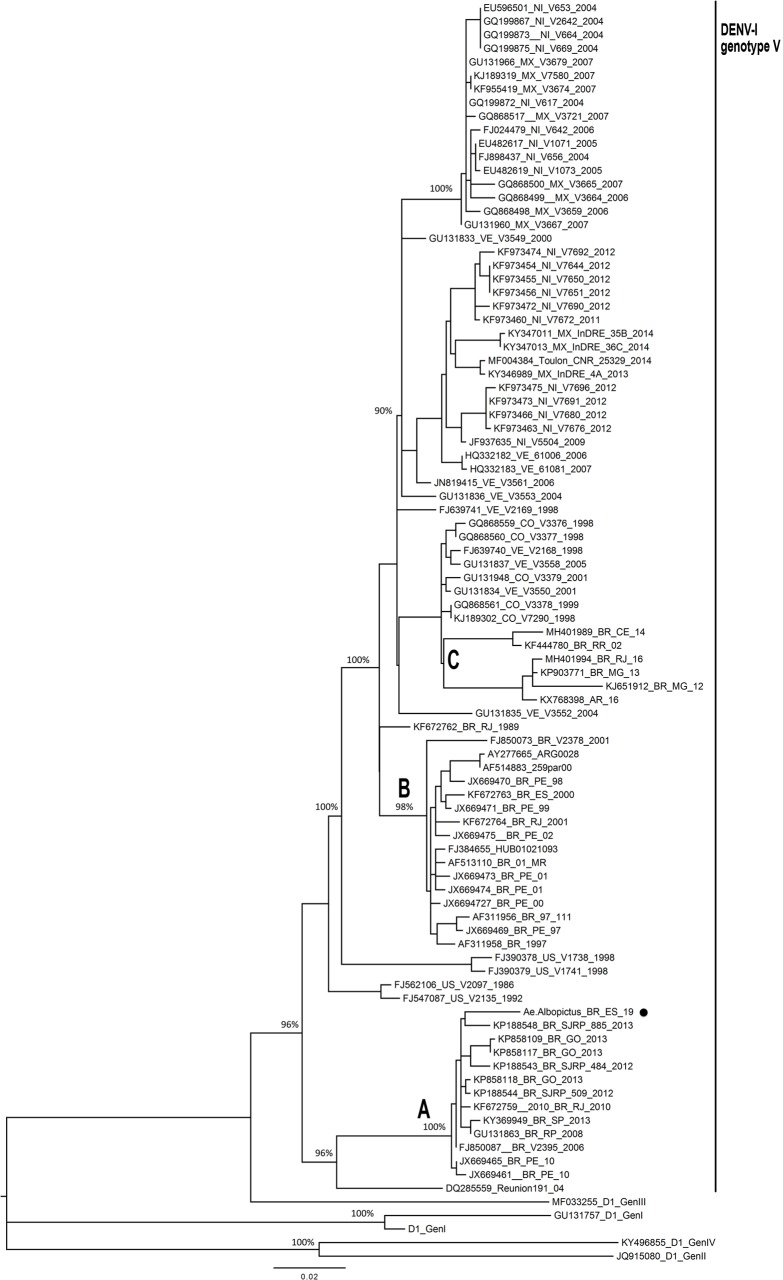
Maximum likelihood phylogenetic tree of the DENV-1 envelope region. *Aedes albopictus* isolate is highlighted and clustered with other Brazilian human viruses sampled in different states. Bootstrap support values of the main nodes are depicted, including the values for clades A, B, and C. Scale bar represents substitutions per site.

The envelope gene of the DENV-1 was successfully amplified and sequenced. The reconstructed phylogenetic tree shows that DENV-1 found in the study belongs to genotype V (Fig 2, clade A). This virus was closely related to strains identified in other Brazilian states in previous years: São José do Rio Preto, in 2012 and 2013, São Paulo in 2013, Goiânia in 2013, Rio de Janeiro in 2010, and Pernambuco in 2010, with 100% bootstrap support. This clade shares a most recent common ancestor (MRCA) with a sample obtained in Réunion, an island in the Indian Ocean. Another two Brazilian clades were identified: Clade B basal to all another DENV-1 genotype V (except by the clade previously mentioned and four samples from Puerto Rico), and clade C close related to Colombian and Venezuelan strains (Fig 2).

## Discussion

This study is the first to report *A*. *albopictus* infected by DENV and ZIKV during an outbreak of a dengue-like illness in a rural area in Brazil. In Brazil, *A*. *aegypti* was previously the only species proven to be involved in the transmission of these viruses, considered typical of urban settings. Despite a tendency of expansion of DENV to the countryside, all the explanations for this phenomenon remain on the establishment of *A*. *aegypti* in smaller cities [39].

Differential diagnosis between DENV and ZIKV infections is challenging due to their similar signs and symptoms [40]. In the rural area under investigation, 20 people presented an exanthematous febrile illness and four had laboratory confirmation for DENV infection. One infection by DENV-1 was identified, and the same serotype was found in *A*. *albopictus*. Despite the absence of confirmed cases of ZIKV infection in humans, six human cases of febrile illness were considered to be not caused by DENV and could have plausibly been a result of ZIKV infection or diseases with similar symptoms, such as those caused by other arboviruses not investigated, e.g. Chikungunya, Mayaro fever and yellow fever. A subsample of mosquitoes infected by ZIKV was engorged, indicating the potential involvement of *A*. *albopictus* in a silent transmission of this virus, despite it is not possible to know if the virus was originated from the human blood or from the mosquito. Therefore, it requires further investigation, also because only the presence of the virus in the mosquito is not enough to confirm its ability to transmit the pathogen to humans and due to this vector feed on non-human hosts in the sylvatic environment. Despite the lack of molecular detection of ZIKV or other arboviruses in the studied human cases, the assumption on the participation of *A*. *albopictus* in the transmission is credible, due to the absence of *A*. *aegypti* even after an extensive entomological sampling. *A*. *albopictus* normally feeds in the daytime and outdoor but can rest and feed indoors [41]. In the study setting, the adult mosquitoes were present during daytime, concentrated in all areas surveyed, corroborating the pattern of the population aggregate distribution, with mosquitoes concentrating in close areas [16].

The DENV-1 genotype V found in the infected *A*. *albopictus* was also detected in previous Brazilian studies [42–44]. The strain from the study setting is closely related to viruses that have circulated previously in large urban centers of three different regions in Brazil (Southeast, Northeast, and Midwest), showing the capacity of the virus dispersion from urban to rural areas. In addition, it demonstrates that the strain found in the *A*. *albopictus* was not new in Brazil, being involved previously in epidemics related to *A*. *aegypti*, suggesting the potential to *A*. *albopictus* in transmitting viruses already circulating in the Brazilian population.

The DENV-1 found in the study had a different origin from a strain identified in 2000 in Espírito Santo state, evidencing multiple introductions of this virus into the state. The clade that contains the strain identified in the *A*. *albopictus* clustered with a virus from Réunion, an island from the Indian Ocean, while the DENV-1 virus circulating in 2000 clustered with viruses from other South American countries. Besides these two clades, the results suggest another clade of DENV-1 in Brazil, closely related to strains from Caribbean countries. It corroborates a most recent study on the phylogenetic evaluation of DENV-1 in the country [44], which suggests at least three clades of this virus in the Brazilian territory.

The introduction of DENV-1 in the rural area under evaluation was related to an outbreak with potential infectivity, raising questions on possible similar future occurrences in Brazil. In this country, previous studies identified field-collected immature forms of *A*. *albopictus* infected by all DENV serotypes [6, 17, 19, 45–47], and by ZIKV [48]. Despite the variation on the vector competence of *A*. *albopictus* according to its geographic origin [49], in other countries this is the only species involved in arboviruses transmission [19, 50], including endemic occurrence in rural areas [5], reinforcing the plausibility of a similar event in Brazil.

A possible scenario of *A*. *albopictus* involvement in DENV and ZIKV transmission in Brazil imposes a concern on the transmission increases, on the establishment of a bridge between sylvatic, rural and urban cycles [15, 51, 52], and on the maintenance of the virus in the environment in non-epidemic periods [5]. In the face of some characteristics of this vector, such as the reports its resistance to some insecticides [9, 11, 12], the use of natural and artificial breeding sites [24], and the broad distribution with ecological plasticity, a possible “ruralization” of DENV and ZIKV may impose additional challenges for the control of these viruses.

This study presents some limitations: it was not possible to identify if the mosquitoes positive for DENV and ZIKV were collected in intradomicile, peridomicile, or in the cocoa and rubber tree plantation, and no inference could be made on it. The insect sampling, restricted to daytime, excluded mosquitoes with nighttime behavior that could be competent vectors for arboviruses, such as *Culex*. In addition, it was not possible to evaluate the origin of the infection of the mosquitoes or the similarity of the virus found in the study with those from the confirmed human infections. The study presents two concomitant events—humans and mosquitoes infected—that might be connected, but it cannot affirm with total certainly the involvement of *A*. *albopictus* in the transmission. Therefore, additional investigations involving the human hosts affected by this outbreak need to be conducted. Nevertheless, the study findings are relevant to the adoption of actions for prevention and control. *A*. *albopictus* must be considered in areas with arbovirus risk transmission and should be included in public health programs, especially with a focus on epidemiological and entomological surveillance, inclusive in rural areas.
